# Alternative and Synergistic Strategies Against Antibiotic‐Resistant *Staphylococcus aureus* in Clinical and Environmental Settings

**DOI:** 10.1155/ijm/8281541

**Published:** 2026-10-16

**Authors:** Kiandokht Babolhavaeji, Sima Kazemi, Tahere Etesamifard, Rahmat Solgi

**Affiliations:** ^1^ Department of Medical Microbiology School of Medicine Shahid Beheshti University of Medical sciences Tehran Iran sbmu.ac.ir; ^2^ Infectious Disease Research Center Avicenna Institute of Clinical Sciences Avicenna Health Research Institute Hamadan University of Medical Sciences Hamadan Iran umsha.ac.ir; ^3^ Autism Spectrum Disorders Research Center Institute of Neuroscience and Mental Health Hamadan University of Medical Sciences Hamadan Iran umsha.ac.ir

**Keywords:** alternative therapy, AMR *S. aureus*, fomites, foods, hospital

## Abstract

Antimicrobial resistance (AMR) in *Staphylococcus aureus* (*S. aureus*) poses a serious global health threat, undermining treatment outcomes and necessitating the development of innovative therapeutic strategies. Methicillin‐resistant *Staphylococcus aureus* (MRSA) is a major contributor to this burden, with increasing prevalence in hospitals, contaminated food, and environmental sources. The coronavirus disease 2019 (COVID‐19) pandemic further intensified *S. aureus* resistance due to widespread antibiotic misuse. Biofilm formation, which facilitates the horizontal transfer of resistance genes such as *mecA* (methicillin resistance gene A), plays a central role in the development of resistance. To counteract antibiotic‐resistant *S. aureus*, alternative approaches have emerged, including antimicrobial peptides (AMPs), photodynamic therapy (PDT), and drug repurposing guided by artificial intelligence (AI). Cutting‐edge technologies such as clustered regularly interspaced short palindromic repeats (CRISPR)‐CRISPR‐associated protein 13a (Cas13a)‐based gene editing and engineered bacteriophages offer promising avenues for targeting resistant strains. Natural compounds such as thymol and chlorogenic acid (CGA) have also shown potential in reducing resistance and inhibiting biofilm formation. Effective management of AMR requires strengthened antibiotic stewardship programs (ASPs), robust surveillance systems, and public health measures such as proper hand hygiene and environmental sanitation. Combining antibiotic adjuvants (AAs) with existing antibiotics and leveraging bioactive peptides (BAPs) can enhance antibiotic efficacy against multidrug‐resistant (MDR), extensively drug‐resistant (XDR), and pan‐drug‐resistant (PDR) *S. aureus*. This review investigates the prevalence and resistance mechanisms of *S. aureus* in clinical, food, and environmental contexts, explores promising alternative therapies, and emphasizes the urgent need for global antimicrobial stewardship (AMS) and sustainable intervention strategies.

## 1. Introduction

Antibiotic resistance in *Staphylococcus aureus* (*S. aureus*), particularly methicillin‐resistant *S. aureus* (MRSA), poses a serious threat to global public health. Similar to other resilient pathogens, *S. aureus* can reduce the efficacy of conventional antimicrobial agents through evolving resistance mechanisms [1, 2]. The emergence of resistance, even among newly developed or recently introduced antibiotics has challenged treatment protocols and limited therapeutic options [3]. Alarmingly, overlapping resistance profiles have been reported between long‐established and newly synthesized antimicrobial compounds, suggesting the adaptability of *S. aureus* under antibiotic pressure [4].

Effective containment of *S. aureus* resistance requires a comprehensive understanding of its ecological distribution and transmission. Although *S. aureus* has traditionally been associated with healthcare settings, numerous studies have reported its presence in food items, livestock, and various community surfaces [5–7]. Environmental surfaces and fomites such as hospital beds, medical equipment, clothing, and personal devices (e.g., mobile phones) serve as potential reservoirs for antibiotic‐resistant *S. aureus* [8, 9]. These contaminated objects play a critical role in the transmission cycle, particularly in healthcare environments. Globally, antimicrobial resistance (AMR) is more frequently reported in countries with lower socioeconomic resources, likely due to limited access to regulated antibiotics and weaker healthcare infrastructures [10]. Nonetheless, resistance is also escalating in high‐income nations, emphasizing the need for coordinated global action [11]. In 2012, Magiorakos et al. [12] proposed standardized definitions for classifying resistance: multidrug‐resistant (MDR), extensively drug‐resistant (XDR), and pan‐drug‐resistant (PDR) strains. XDR and PDR forms of *S. aureus*, particularly those isolated in hospitals, complicate infection control and are often associated with MRSA [13]. Although antibiotics remain crucial in managing *S. aureus* infections, overuse and misuse have contributed significantly to resistance development [14]. To address this, nations like China have implemented nationwide plans to combat AMR, including the National Action Plans spanning 2016–2020 and 2022–2025 [15]. Similarly, stewardship initiatives aim to curb irrational antibiotic prescriptions and improve infection prevention strategies [16]. Notably, the prevalence of MRSA has risen in several regions, including the United Arab Emirates [17] and Finland [18], highlighting the persistent burden on healthcare systems. A 2021 report by the Global Research on Antimicrobial Resistance (GRAM) estimated that 4.71 million deaths globally were associated with bacterial resistance, projecting further healthcare and economic consequences by 2050 [18]. Given the mounting burden of AMR, this review focuses on the occurrence of resistant S. aureus strains in food products, fomites, and hospital settings. We also summarize recent advances in alternative therapies including photodynamic therapy (PDT), bacteriophage applications, nanoconjugates, and antibiofilm agents that offer potential solutions for managing AMR *S. aureus* in an era of dwindling antibiotic efficacy.

## 2. Environments Commonly Associated With AMR *S. aureus*

AMR *S. aureus* has been identified in diverse environments, yet its prevalence in contaminated food remains underreported in the literature [19]. This review aims to highlight the food‐related sources where AMR *S. aureus* has been detected. One significant route of contamination involves human carriers; *S. aureus* is commonly found as part of the nasal and skin microbiota of food handlers, including street food vendors, making direct contact a likely source of transmission [20, 21]. This issue extends beyond individuals to food‐contact surfaces and dairy products, where resistance genes can be disseminated through shared equipment and fomites [19, 22, 23]. The presence of AMR *S. aureus* in food systems underscores the need for stricter hygiene and safety measures during food processing and handling [24]. Interestingly, a study by Bali et al. [25] analyzing isolates from water, soil, surfaces, and air found no statistically significant difference in virulence factor expression between MDR and antibiotic‐sensitive *S. aureus*, suggesting that resistance may not always correlate with virulence. Importantly, hospital‐prepared meals despite being produced under stringent sanitary conditions have also been shown to harbor AMR *S. aureus*, raising concerns about cross‐contamination even in controlled settings [26]. Ready‐to‐eat foods, due to their minimal handling before consumption and widespread public use, pose a particularly high risk if contaminated [27]. Contamination may occur through food workers′ hands during cooking or serving, facilitating AMR gene transmission [28]. Kitchens serve as critical nodes for AMR transmission, where improper handling can result in cross‐contamination between raw and cooked foods for example, through shared utensils or surfaces, sustaining *S. aureus*′s resistance in the food chain [29]. Additionally, occupational exposure has been implicated in *S. aureus* transmission; for instance, nasal carriage of MRSA has been documented among poultry workers and butchers in Nigerian slaughterhouses, emphasizing the need for protective measures in animal‐handling professions [30]. Table 1 summarizes various food items and food‐related environments where AMR *S. aureus* contamination has been reported.

**Table 1:** List of some AMR *S. aureus* isolated from foods.

Source	Region	Time of collection	Antibiotic resistance phenotype	Reference
Cattle and buffalo milk	Dairy farms in Sharkia and Dakahlia, Egypt	2018–2020	Vancomycin, aminoglycosides, fluoroquinolones, cephalosporins, ansamycin, fucidanes, glycopeptides, glycylcyclines, lincosamides, lipopeptides, macrolides, nitrofurans, oxazolidinones, penicillin + beta‐lactamase inhibitors, phenicols, phosphonic acid, polypeptides, streptogramins, sulfonamides, tetracyclines	[31]
Nile Tilapia fish (*Oreochromis niloticus*)	Nairobi, Kenya	—	Tetracyclines, beta‐lactams, sulfonamides, trimethoprim	[32]
Cow quarter milk, bulk tank milk, dogs	Farms in Vermont, United States	—	Beta‐lactams	[33]
Foods including minced meat, butter, raw milk, pastries	Tizi Ouzou area, Algeria	2015–2019	Penicillins, tetracyclines, cefoxitin, ofloxacin, erythromycin, chloramphenicol	[34]
Beef, milk	Butchery and milk markets in Faisalabad, Pakistan	2022	Ceftriaxone, cefepime, co‐amoxiclav, ciprofloxacin, ofloxacin, tetracycline, tobramycin, gentamicin, trimethoprim‐sulfamethoxazole, meropenem, imipenem, penicillin, erythromycin, cefoxitin, clindamycin	[35]
Mixed foods, milk and dairy products, meat, fish, vegetables, juices	European union	2021	Penicillins, beta‐lactams, aminoglycosides, macrolides, lincosamides, streptogramin B, fluoroquinolones, vancomycin, linezolids, trimethoprim	[24]
Egg	Indonesia	5‐year study	Tetracycline, ampicillin, amoxicillin, ciprofloxacin, ceftriaxone, beta‐lactam, azithromycin	[36]
Ready‐to‐eat meat products	Kabul, Afghanistan	January–May 2023	Chromphenicol, levofloxacin, ciprofloxacin, erythromycin, tetracycline, oxacillin	[28]
Milk and milk products	Ambo and Bako towns, Oromia, Ethiopia	—	Amoxicillin, oxacillin, ampicillin, cefoxitin, tetracycline	[37]
Raw cow milk	Meta district of eastern Hararghe zone, Oromia, Ethiopia	December 2018–August 2019	Erythromycin, cefoxitin, streptomycin, vancomycin, penicillin, tetracycline, amoxicillin, ampicillin, sulfamethoxazole, kanamycin	[38]
Cherry tomato, pear, lettuce	Shanghai, China	June–September 2021	Oxacillin, ampicillin, penicillin (SCC‐mec IV)	[39]
Milk, cottage cheese, and yogurt	Arsi highlands, Oromia, Ethiopia	March 2022–February 2023	Beta‐lactams (penicillin G, ampicillin, amoxicillin‐clavulanic acid), tetracycline, sulfonamides (trimethoprim‐sulfamethoxazole), cephosporines (cefoxitin), phenicols (chloramphenicol), lincosamides (clindamycin)	[40]
Raw milk	Shanghai, China	2013–2022	Ofloxacin, erythromycin, cefoxitin, entrofloxacin, tilmicosin, clindamycin, penicillin, trimethoprim‐sulfamethoxazole, amoxicillin‐clavulanic acid, sulfamethoxazole	[41]
Pigs	Germany	2012–2021	Plasmid‐coded linezolide	[42]

## 3. AMR *S. aureus* in Hospital Settings

*S. aureus* is among the most frequently isolated nosocomial pathogens, particularly from blood specimens of patients diagnosed with bacteremia [43]. Bacteremia represents one of the most serious complications of MDR and XDR *S. aureus* infections, especially in immunocompromised populations such as cancer patients, where the presence of resistant strains significantly increases morbidity and mortality rates [44, 45]. Both MDR and XDR *S. aureus* strains have also been isolated from diabetic foot infections, with most cases classified as MDR. Notably, the majority of MDR isolates were MRSA, followed by extended‐spectrum beta‐lactamase (ESBL)‐producing strains [46]. Although PDR isolates are less frequently reported, their presence highlights the critical need for robust antimicrobial stewardship programs. Clinical studies show that PDR *S. aureus* strains are less common than XDR, and XDR less common than MDR, reflecting the progressive accumulation of resistance traits [47, 48]. The persistence of *S. aureus* in hospital environments is driven by its ability to undergo adaptive changes during colonization, allowing prolonged survival in host tissues [49]. Hematogenous dissemination of *S. aureus* from bacteremia may result in secondary infections, such as periprosthetic joint infections [50]. Table 2 summarizes the characteristics of antibiotic‐resistant *S. aureus* isolates from hospital‐ and community‐acquired infections.

**Table 2:** Characteristics of antibiotic resistant *S. aureus* isolates from either hospital or community‐acquired infections.

Patients characteristics	Source of infection	Hospital ward	Sample type	Location	Time of collection	Prevalent antibiotic resistance	Reference
Age groups: 0–15, 16–40, **41–65** ^**a**^, 66 ≤	Nosocomial for *S. aureus* nasal carriers in surgical department and ICU patients	**Surgery**, internal medicine, ICU, infectious	**Pus**, specimens from respiratory tract, blood and other sterile body fluids, urine, stool	General hospital, Vietnam	January 2014–December 2021	Azithromycin, erythromycin, clindamycin, cefoxitin	[51]
Cancer (**breast**, **cervical**, leukemia, colorectal, osteosarcoma, stomach, liver, pancreas, prostate, kaposi sarcoma, cavum, lung, cholangiocarcinoma, ovary, esophagus) and noncancer patients	Nosocomial	ICU	**—**	Laquintinie hospital, Douala, Littoral region, Cameroon	October 2021–March 2023	Cefoxitin, amoxicillin, ceftazidime, fusidic acid, tetracycline, erythromycin, trimethoprim‐sulfamethoxazole (both cancer and noncancer patients), nitrofurantoin, ofloxacin, amoxicillin‐clavulanic acid in cancer patients	[52]
—	Nosocomial	—	**Sputum**, urine, blood, secretions	China	January 1, 2022–June 30, 2023	Penicillin, erythromycin, clarithromycin, clindamycin, azithromycin	[53]
Patients aged **60 ≤**	Nosocomial for patients in critical care department.	**Critical care medicine, dermatology**	**Sputum**	Hebei, China	January 2018–December 2022	Increasing tetracycline resistance, decreasing erythromycin and clindamycin resistance	[54]
—	Community for skin and burn infections	**Dermatology**, **orthopedic**,**endocrinology**, **burn**, pediatric, nephrology, oncology, otolaryngology head and neck surgery, neuro‐surgery, vascular surgery, obstetric, thyroid hernia surgery, breast surgery	Skin and soft tissue	Southern Jiangxi, China	2023	oxacillin, penicillin G, cefoxitin, clindamycin, erythromycin	[55]
Children aged **<** 5 years with bacteremia	Community	**—**	Blood	Manhica district hospital, southern Mozambique	January 1, 2001–December 31, 2019	Penicillin, tetracycline	[56]
—	—	—	**Blood**, wound, ocular, ear, sputum, bronchial aspirates, bronchoalveolar lavage fluid	Tertiary care hospital, Romania	January 1, 2017–December 31, 2022	Penicillin, oxacillin, erythromycin, tetracycline, clindamycin inducible resistance	[13]

*Note:*“—” not mentioned.

Abbreviation: ICU, intensive care unit.

^a^Prominent cases are bolded.

During the COVID‐19 pandemic, increased antibiotic use correlated with a rise in antibiotic‐resistant *S. aureus* strains. A study by de Oliveira Whitaker et al. reported that previous azithromycin administration in COVID‐19 patients with bloodstream infections was associated with *S. aureus* isolates resistant to erythromycin, clindamycin, and cefoxitin [57]. The adaptability of *S. aureus*′s genome has been implicated in its persistence in hospital environments. The genomic plasticity of MRSA facilitates the sequential acquisition of AMR genes, complicating treatment options [58, 59]. Nonnasal colonization sites also appear important in healthcare settings. Chmielewski et al. found higher MRSA prevalence in oral samples compared with nasal swabs, and genetic analysis revealed differing strain origins between the two sites despite their anatomical proximity [60]. Because AMR *S. aureus* commonly colonizes human skin, transmission via direct contact between patients and healthcare personnel is a significant concern [61]. Additionally, fomites (e.g., medical instruments, surfaces, bedding) play a critical role in nosocomial transmission and contribute to hospital‐acquired infections [62, 63].

## 4. Beyond Antibiotic Usage

Although linezolid and rifampin have been shown to be among the most effective antimicrobial agents against MRSA, the growing resistance among *S. aureus* isolates has undermined global efforts to treat related infections [14, 64]. Therefore, complementary or alternative therapeutic strategies are urgently required to combat antibiotic‐resistant *S. aureus* [65]. Drug repurposing is currently undergoing clinical trials, and artificial intelligence (AI), including machine learning algorithms, has been employed to predict resistance patterns and guide personalized antimicrobial therapy [66, 67]. Additionally, next‐generation sequencing (NGS) allows for tracking the transmission of antibiotic‐resistant *S. aureus*, although limitations in data accessibility can restrict its widespread application [67, 68]. Novel approaches targeting resistance mechanisms have shown promise. For example, significant downregulation of efflux pumps has been observed with chlorogenic acid and cornosol treatment, offering a strategy to tackle MDR and XDR *S. aureus* [69]. However, this evidence is currently limited to in vitro studies with small sample sizes, and no animal or clinical data are yet available to confirm in vivo efficacy. Furthermore, the bioavailability and metabolic stability of these natural compounds remain poorly characterized, representing a significant barrier to clinical translation. Chitosan‐loaded formulations of *Lagenaria siceraria* and *Thymus vulgaris* have demonstrated antimicrobial and antioxidant properties against vancomycin‐resistant *S. aureus* strains [70]. These findings are derived from in vitro assays and a limited number of in vivo experiments; larger animal studies and eventual clinical trials are needed to establish safety, optimal dosing, and long‐term efficacy. Potential toxicity of chitosan‐based nanoparticles to host tissues has not been comprehensively evaluated. Furthermore, Iturin AC15, a cyclic lipopeptide extracted from *Enterobacter cloacae* associated with *Prosopis cineraria* roots, disrupts MRSA cell membranes [71]. This evidence is primarily in vitro, with no published in vivo or clinical validation to date. The narrow spectrum of activity, potential hemolytic activity, and stability issues of lipopeptides under physiological conditions remain unresolved challenges that must be addressed before clinical application. Biofilm formation is a key contributor to antibiotic resistance in MRSA [72]. Biofilms provide a niche that facilitates horizontal gene transfer, such as the spread of the mecA gene via natural transformation [73]. Disruption of MRSA biofilm has been successfully achieved by combining oxytetracycline (OTC) with calcium gluconate (CaG) [74]. However, this evidence is largely derived from in vitro biofilm models, with limited in vivo validation. The sample sizes in existing studies are small, and the long‐term stability of this combination, as well as its potential to induce resistance, has not been systematically assessed. PDT has emerged as a promising nonantibiotic strategy. Thymol, a phenolic compound from thyme oil, combined with blue‐laser irradiation, increases reactive oxygen species (ROS) levels, leading to oxidative stress and rapid killing of biofilm‐associated MDR *S. aureus*. Blue‐laser activates thymol to its reactive forms, thymoquinone and thymohydroquinone [75]. Although these findings are promising, the evidence is predominantly in vitro, with only limited in vivo validation in animal models. Key limitations include restricted light penetration into deep tissues, dependence on specialized laser equipment, and potential phototoxicity to host cells. Furthermore, standardization of light dose and photosensitizer concentration remains a significant barrier to clinical translation. Similarly, Gao et al. demonstrated that a silver‐palladium bimetallic alloy (AgPd0.38) mimics oxidase activity, generating surface‐bound ROS that coat and kill *S. aureus* without promoting adhesion [76]. This evidence is primarily in vitro, and the biocompatibility, long‐term stability, and potential environmental impact of silver‐palladium nanoparticles require further investigation before clinical use. Laser irradiation has also been applied to enhance the antibacterial effects of thioridazine. Tozar et al. showed that this method outperformed ciprofloxacin in eliminating MRSA [77]. However, this evidence is limited to in vitro studies with small sample sizes, and no animal or clinical data are available. The potential for thioridazine photoproducts to induce toxicity in host cells and the feasibility of delivering laser light to deep‐seated infections remain unresolved. Curcumin, a natural compound with antimicrobial activity, also acts as a photosensitizer. When used in PDT, it significantly reduced the minimum inhibitory concentration (MIC) of multiple antibiotics against resistant *S. aureus*, and decreased phenotypic heterogeneity [78]. Although curcumin has the advantage of being a natural, relatively safe compound, its poor water solubility, low bioavailability, and rapid metabolism limit its clinical utility. Most studies are in vitro, and larger in vivo studies are needed to confirm efficacy and safety.

Rhamnolipid, a biosurfactant from *Pseudomonas aeruginosa* MA01, increases MRSA membrane permeability and reduces biofilm formation, as confirmed by flow cytometry [79]. This evidence is primarily in vitro, with limited in vivo validation. The potential cytotoxicity of rhamnolipids to host cells, their stability in physiological environments, and the risk of promoting *Pseudomonas* coinfections are important concerns that require further investigation. Similarly, lipidic extracts from microalgae have effectively photosensitized antibiotic‐resistant *S. aureus* [80]. These findings are in vitro, and the complex composition of microalgal extracts poses challenges for standardization and quality control. In vivo studies are needed to assess efficacy and safety. Recognizing *S. aureus* as a food contaminant, Hou et al. developed the Qu/Ce6@ZIF‐8 nanoplatform to enhance PDT efficacy [81]. This nanoplatform has shown promising in vitro results, but in vivo validation in food matrices and animal models is still lacking. Potential toxicity of the nanoplatform to human cells and its environmental impact require thorough evaluation. APTES‐modified zinc oxide quantum dots (ZnO‐QDs) have also shown strong anti‐MRSA activity without inducing resistance [82]. Although these results are encouraging, the evidence is predominantly in vitro, and concerns regarding the long‐term toxicity, biocompatibility, and environmental persistence of ZnO‐QDs remain to be addressed. Proteomic tools have been employed to assess protein expression in *S. aureus*. Erythrosine‐mediated PDT reduced *S. aureus* to undetectable levels [83]. This evidence is primarily in vitro, and although erythrosine is FDA‐approved as a food colorant, its efficacy as a photosensitizer in clinical settings, optimal dosimetry, and potential for inducing resistance require further study. Quorum sensing (QS) plays a central role in regulating MRSA virulence and resistance. Bioactive peptides from two lactic acid bacteria strains sensitized MRSA to cefoxitin, suggesting that these metabolites may enhance *β*‐lactam efficacy [84]. This evidence is primarily in vitro, with limited in vivo validation. The stability of these bioactive peptides in physiological conditions, their potential immunogenicity, and the risk of disrupting beneficial microbiota are important considerations for clinical translation. Biofilm dispersal is influenced by antibiotic pharmacokinetics, and adjuvants can enhance antibiotic efficacy [85]. Salicylic acid, an aspirin metabolite, disrupts QS (specifically agr), thereby inhibiting biofilm dispersal. Coadministration of aspirin with antibiotics improves their effectiveness against resistant *S. aureus* [86]. Although aspirin is a well‐established, safe drug, the evidence for its synergistic effect with antibiotics is primarily in vitro and in animal models. Clinical trials are needed to determine optimal dosing, potential drug interactions, and long‐term safety, particularly in patients with contraindications to aspirin.

Phage therapy represents another promising alternative. Bacteriophages are practical, cost‐effective, and can be engineered for targeted activity, although clinical standardization is lacking [87]. The evidence for phage therapy spans in vitro, in vivo (including zebrafish embryo models), and sporadic clinical case reports; however, sample sizes in clinical studies are extremely small (*n* = 1–5), limiting generalizability. Key limitations include host immune responses to phages, narrow host range, and the potential for bacterial resistance to phages. Barriers to clinical translation include the lack of a standardized regulatory framework, difficulties in mass production, and quality control challenges. Engineered phages can be converted into phagemids or phasmids [88]. A CRISPR‐Cas13a‐equipped phagemid targeting mecA has shown promising results in eradicating MRSA [89]. This evidence is primarily in vitro and in animal models, with very limited clinical data. Challenges include efficient delivery of the phagemid to target bacteria, potential off‐target effects, and immune responses. Standardization and large‐scale production remain significant hurdles. In another strategy, H_2_S scavengers have been used to counter bacterial defense mechanisms, restoring gentamicin efficacy though this effect is neutralized by exogenous H_2_S [90]. This evidence is primarily in vitro and in animal models, and the specificity, safety, and long‐term effects of H_2_S scavengers in humans are not yet established. Phage therapy has even been explored using zebrafish embryo models for MRSA treatment [91, 92]. Although zebrafish models offer valuable insights, they do not fully recapitulate human physiology and immune responses; therefore, mammalian models and eventual clinical trials are essential. Antimicrobial peptides (AMPs), such as KTA and KTR, inhibit peptidoglycan synthesis and permeabilize the *S. aureus* cell wall [93]. The evidence for AMPs is primarily in vitro and in vivo (mouse models), with variable sample sizes (*n* = 5–20). Key limitations include potential host cell toxicity (e.g., hemolysis), susceptibility to proteases, and low stability. Barriers to clinical translation include high production costs, difficult formulation, and short half‐life. Hybrid peptide systems specifically targeted AMPs combined with bacterial‐trapping peptides exhibit high efficacy against MRSA [94]. These findings are primarily in vitro and in animal models; clinical validation is needed. Potential immunogenicity and long‐term safety of hybrid peptides remain to be established. Peptide adjuvants like 3‐WP and 9‐PR enhance the antibiofilm activity of melittin, aiding both infection control and food preservation [95]. This evidence is primarily in vitro, with limited in vivo validation. The safety of melittin and its adjuvants for human use, particularly regarding hemolytic activity, requires thorough investigation. Epifadin, a bacteriocin produced by nasal *Staphylococcus epidermidis*, eradicates nasal *S. aureus* colonization [96]. This evidence is primarily in vitro and in animal models; clinical studies are needed to confirm efficacy and safety in humans. Potential disruption of the nasal microbiome and long‐term effects on colonization resistance are important considerations. Additionally, combining bacteriocins garvicin KS and micrococcin P1 effectively compromises MRSA cell viability [97]. This evidence is primarily in vitro, and the narrow spectrum, stability issues, and potential for resistance development need to be addressed before clinical application.

## 5. Concluding Remarks and Future Direction

AMR remains one of the leading causes of mortality worldwide, particularly in low‐resource countries. Conventional therapies are becoming increasingly ineffective against AMR *S. aureus* infections, highlighting an urgent need for alternative treatment strategies. The rapid emergence and dissemination of AMR *S. aureus* pose significant challenges to the success of current antimicrobial therapies. This growing threat underscores the importance of sustained genomic and epidemiological surveillance to monitor the burden of AMR and to prevent a looming public health crisis. Moreover, AMR *S. aureus* is not only a healthcare‐associated pathogen but also a foodborne contaminant. Enhancing food safety guidelines, especially in food processing and cooking practices, is crucial to reduce exposure risks. In healthcare settings, *S. aureus* persists on high‐touch surfaces such as light switches and doorknobs, acting as reservoirs for nosocomial infections. The persistence of AMR S. aureus in clinical environments necessitates strict hygiene measures. These include regular handwashing and environmental sanitization by healthcare workers significant advancements have been made in developing targeted policies to better understand and manage this pathogen. To improve antibiotic efficacy, novel adjuvants and alternatives including sequence‐based bactericidal agents—have been introduced. These innovative approaches offer promising solutions to overcome the challenges posed by XDR and PDR *S. aureus*, emphasizing the importance of tailored antibiotic prescriptions based on local resistance trends.

The continued rise of antimicrobial‐resistant *S. aureus* in the clinical, environmental, and food environments is a dynamic and multifaceted public health issue. Traditional antibiotics alone are insufficient. An interdisciplinary approach is required, integrating next‐generation technologies, novel therapeutics, and enhanced infection control procedures. This review identifies a shared repertoire of novel strategies spanning CRISPR‐mediated gene editing, PDT, bacteriophage therapy, to AMPs and quorum sensing inhibitors—that together delineate the promising directions to tackle MDR, XDR, and PDR *S. aureus*. Upcoming studies must be directed towards the standardization and translation into the clinic of new therapeutic modalities like phage engineered, AI‐augmented drug repurposing, and AMPs for repeated and safe use in humans. Resistance pattern longitudinal surveillance programs are required to track hospital, community, and food industry resistance patterns. Combination therapy testing with adjuvants or natural products is essential to regain the effectiveness of current antibiotics. One Health systems must be expanded to better tackle zoonotic and foodborne modes of transmission. Biofilm‐directed strategies, such as nanoparticle‐conjugates and biofilm‐dispersal agents, must be explored in investigating the eradication of persistent colonization and relapse. Finally, collaborative interdisciplinary effort and investment in translational science are needed in a bid to match the adaptive capacity of *S. aureus* and counter the global threat occasioned by AMR.

**Nomenclature**
AMPs: antimicrobial peptides
AMR: antimicrobial resistant/resistance
MDR: Multidrug resistant
MRSA: methicillin resistant *Staphylococcus aureus*
PDR: pan‐drug resistant
XDR: extensively drug resistant

## Author Contributions

K.B. conceived the main idea, wrote the manuscript, and designed the tables. S.K., T.E., and R.S. edited the manuscript.

## Funding

No funding was received for this manuscript

## Disclosure

All authors read and approved the final manuscript.

## Consent

The authors have nothing to report.

## Conflicts of Interest

The authors declare no conflicts of interest.

## Data Availability

The data that support the findings of this study are available from the corresponding author upon reasonable request.
